# From Mood to Memory: Unlocking Saffron’s Potential in Brain Health

**DOI:** 10.7759/cureus.82924

**Published:** 2025-04-24

**Authors:** Tarlan Kehtari, Diego Cardenas Tovar, Dustin Epstein, Patricia Junquera

**Affiliations:** 1 Medicine, Herbert Wertheim College of Medicine, Florida International University, Miami, USA; 2 Psychiatry and Behavioral Sciences, Herbert Wertheim College of Medicine, Florida International University, Miami, USA

**Keywords:** alzheimer's disease, anxiety disorders, cholinesterase inhibitors, crocus sativus, depression treatment, mild cognitive impairment, neurodegenerative disease, neuroinflammation, saffron, selective serotonin reuptake inhibitor (ssri)

## Abstract

Depression, anxiety, mild cognitive impairment (MCI), and Alzheimer’s disease (AD) pose ongoing therapeutic challenges, particularly in aging populations. Conventional pharmacologic treatments often have delayed onset, limited efficacy, and unfavorable side effects, prompting interest in natural alternatives. Saffron (*Crocus sativus* L.), a spice long used in traditional medicine, has emerged as a promising candidate due to its multimodal neuroprotective properties.

This review evaluated the clinical efficacy, safety, and mechanistic profile of saffron in the treatment of mood and cognitive disorders. A targeted literature search identified multiple double-blind randomized controlled trials (RCTs) and meta-analyses comparing saffron to standard pharmacologic agents or placebo. In depression, RCTs using 30 mg/day of saffron for six weeks showed comparable improvements in Hamilton Depression Rating Scale (HAM-D) scores to fluoxetine (20 mg/day), with significant reductions from baseline and no difference in adverse events. An adjunctive study using crocin (an active ingredient of saffron; 15 mg twice daily) also demonstrated additive effects alongside selective serotonin reuptake inhibitors (SSRIs). In cognitive disorders, saffron (30 mg/day) showed non-inferiority to donepezil (10 mg/day) and memantine (20 mg/day) in patients with mild-to-moderate and moderate-to-severe AD. Trials lasting 16 to 52 weeks demonstrated significant improvements on validated cognitive scales such as Alzheimer's Disease Assessment Scale - Cognitive Subscale (ADAS-Cog) and Clinical Dementia Rating - Sum of Boxes (CDR-SB), with fewer gastrointestinal side effects compared to standard treatments. Meta-analyses confirm the superiority of saffron over placebo and its equivalence to conventional drugs, with no serious adverse events.

Saffron represents a safe, well-tolerated, and culturally integrated intervention with growing evidence for use in psychiatric and neurologic disorders. Larger, multicenter trials with biomarker validation and standardized extract formulations are needed to confirm its role in clinical practice.

## Introduction and background

Neuropsychiatric and neurodegenerative disorders such as depression, mild cognitive impairment (MCI), and Alzheimer’s disease (AD) are among the most prevalent and debilitating conditions worldwide. These disorders frequently coexist and contribute to substantial functional decline, caregiver burden, and healthcare expenditures, particularly in aging populations. Depression and anxiety are not only prevalent in older adults but may also precede AD by decades. These symptoms could represent prodromal manifestations of neurodegeneration or contribute to its development [1-5]. The current first-line therapies often produce limited symptomatic relief, delayed therapeutic onset, and adverse effects such as gastrointestinal discomfort, sedation, and emotional blunting [6,7].

Given these limitations, there has been increasing interest in identifying safe, effective, and culturally integrative treatments with multimodal actions. Saffron (*Crocus sativus* L.), a spice long used in Persian, Mediterranean, and South Asian traditions, has emerged as a promising candidate for mental and cognitive health. Historically incorporated into food and traditional remedies, saffron has attracted modern scientific attention for its potential antidepressant, anxiolytic, and neuroprotective properties [8,9].

Mechanistically, saffron and its active constituents - crocin, crocetin, and safranal - appear to act through several neurobiological pathways. These include serotonergic and dopaminergic modulation, cholinesterase inhibition, antioxidant defense, and anti-inflammatory effects, making it particularly relevant to both mood regulation and cognitive decline [6,8,10]. This is especially important given the bidirectional relationship between depression and dementia, with mood symptoms often preceding or accelerating neurodegenerative changes [11].

Despite encouraging clinical and mechanistic findings, saffron remains underutilized in clinical practice. Challenges include the lack of long-term safety data, standardization difficulties, variability in extract composition, and limited regulatory oversight [6]. Furthermore, few studies have explored patient-specific factors such as pharmacogenomic markers or metabolic profiles that may influence treatment response, which are critical steps toward personalized neuropsychiatric care.

This review synthesizes clinical and mechanistic evidence on saffron’s role in treating depression, MCI, and AD. We focused on these conditions due to their frequent overlap, shared pathophysiology, and growing support from both preclinical and clinical studies. Our goal is to clarify saffron’s therapeutic value and its potential integration into neuropsychiatric care.

## Review

Methods

This literature review was conducted to evaluate the efficacy, safety, and mechanisms of saffron (*Crocus sativus L*.) in treating depression, MCI, and AD. PubMed and Google Scholar were searched using combinations of the following keywords: “saffron,” “Crocus sativus,” “depression,” “anxiety,” “mild cognitive impairment,” and “Alzheimer’s disease.” Only English-language, peer-reviewed human randomized controlled trials (RCTs), systematic reviews, and meta-analyses were included. Studies assessing saffron’s clinical efficacy, safety profile, and biological mechanisms in mood or cognitive disorders were prioritized.

Inclusion criteria consisted of double-blind RCTs comparing saffron to placebo or standard pharmacologic agents such as SSRIs, cholinesterase inhibitors, or N-methyl-D-aspartate (NMDA) receptor antagonists. Trials evaluating saffron as both monotherapy and adjunctive therapy were considered. Exclusion criteria included animal studies, case reports, editorials, and studies lacking control groups or standardized dosing. Data extraction focused on sample size, treatment duration, saffron formulation and dose, comparator interventions, clinical outcomes, effect sizes, and adverse events.

Saffron for depression and anxiety

Multiple RCTs have investigated saffron’s efficacy in treating mild-to-moderate major depressive disorder (MDD) and have consistently demonstrated statistically significant improvements comparable to conventional antidepressants (Table 1).

**Table 1:** Summary of the clinical studies evaluating saffron for mood disorders DB-RCT, double-blind randomized controlled trial; HAM-D, Hamilton Depression Rating Scale; BDI, Beck Depression Inventory; SSRI, selective serotonin reuptake inhibitor; BID, twice daily; SMD, standardized mean difference

Author (Year)	Design	Sample size	Duration	Intervention	Comparator	Primary outcome(s)	Key results
Akhondzadeh et al. (2005) [12]	DB-RCT	40	6 weeks	Saffron 30 mg/day	Fluoxetine 20 mg/day	HAM-D	Comparable reduction in HAM-D; p>0.05
Noorbala et al. (2005) [13]	DB-RCT	40	6 weeks	Saffron 30 mg/day	Fluoxetine 20 mg/day	HAM-D	Significant within-group reduction; no group difference (p>0.05)
Moshiri et al. (2006) [9]	DB-RCT	40	6 weeks	Saffron petal extract 30 mg/day	Placebo	HAM-D	Saffron significantly superior to placebo (p<0.001)
Shahmansouri et al. (2014) [11]	DB-RCT	40	6 weeks	Saffron 30 mg/day	Fluoxetine 20 mg/day	HAM-D	Comparable reductions in HAM-D (p=0.47); effective in cardiac population
Talaei et al. (2015) [10]	DB-RCT (adjunctive)	40	4 weeks	Crocin 15 mg BID + SSRI	Placebo + SSRI	BDI	Greater improvement with crocin (ΔBDI: 21.6 vs. 9.2; p<0.01)
Dai et al. (2020) [7]	Meta-analysis	278 (5 RCTs)	Varied	Saffron 30 mg/day (avg)	SSRIs / Placebo	HAM-D, BDI	Saffron > placebo (SMD = -1.22; p < 0.001); equivalent to SSRIs (p = 0.45)

In one of the earliest trials, Akhondzadeh et al. (2005) conducted a six-week, double-blind RCT involving 40 outpatients diagnosed with MDD. Participants were randomly assigned to receive either saffron capsules (30 mg/day) or fluoxetine (20 mg/day). The Hamilton Depression Rating Scale (HAM-D) was used as the primary outcome measure and both groups exhibited significant improvement from baseline, with no statistically significant difference between them (mean HAM-D reduction: saffron=12.0±5.1; fluoxetine=11.0±4.5; p>0.05), indicating comparable efficacy [12].

A similar six-week RCT by Noorbala et al. (2005) enrolled 40 patients with mild-to-moderate depression, comparing a hydro-alcoholic extract of saffron (30 mg/day) to fluoxetine (20 mg/day). Both treatment arms showed significant reductions in HAM-D scores from baseline, with no significant between-group differences (p>0.05). This study reinforced the hypothesis that saffron may exert antidepressant effects similar to selective serotonin reuptake inhibitors (SSRIs) [13].

Moshiri et al. (2006) extended this evidence by examining saffron derived specifically from petals rather than stigmas. In their six-week RCT (n=40), saffron petal extract (30 mg/day) was again found to be significantly more effective than placebo in reducing HAM-D scores (p<0.001), suggesting that various parts of the plant may possess mood-enhancing properties [9].

Shahmansouri et al. (2014) conducted a six-week, double-blind RCT comparing saffron (30 mg/day) to fluoxetine (20 mg/day) in patients with MDD who had undergone percutaneous coronary intervention (PCI; n=40). Both treatment groups experienced statistically significant reductions in HAM-D scores from baseline (saffron: 24.5 → 11.5; fluoxetine: 23.8 → 10.2), with no significant difference in treatment response (p=0.47), indicating that saffron may be suitable even in medically complex populations [11].

Talaei et al. (2015) explored saffron’s potential as an adjunctive treatment. In their 4-week RCT of patients with MDD already receiving SSRIs (n=40), participants were randomized to adjunctive crocin (15 mg twice daily) or placebo. The crocin group showed significantly greater improvements in the Beck Depression Inventory (BDI) scores than placebo (mean change: 21.6 vs. 9.2, p<0.01), suggesting additive antidepressant effects [10].

A comprehensive meta-analysis by Dai et al. (2020) reviewed five RCTs totaling 278 participants and concluded that saffron significantly reduced depressive symptoms compared to placebo (standardized mean difference (SMD)=-1.22, 95% CI: -1.94 to -0.49, p<0.001) and showed no significant difference when compared with SSRIs (SMD=0.16, 95% CI: -0.26 to 0.59, p=0.45). Importantly, dropout rates and adverse events were similar across treatment arms, underscoring saffron’s tolerability [7].

As for side effects, saffron was generally well tolerated across all trials. Reported adverse events were mild and included nausea, headache, and appetite changes. No serious adverse events or treatment-emergent suicidal tendencies were reported.

Saffron for cognitive disorders

Saffron (*Crocus sativus* L.) has demonstrated promising efficacy in the treatment of AD and MCI, with multiple RCTs and meta-analyses suggesting comparable cognitive outcomes to standard pharmacologic therapies such as donepezil and memantine (Table 2).

**Table 2:** Summary of the clinical studies evaluating saffron for cognitive disorders DB-RCT, double-blind randomized controlled trial; ADAS-Cog, Alzheimer’s Disease Assessment Scale-Cognitive Subscale; CDR-SB, Clinical Dementia Rating-Sum of Boxes; SCIRS, Severe Cognitive Impairment Rating Scale; FAST, Functional Assessment Staging Tool; ADL, Activities of Daily Living.

Author (Year)	Design	Sample size	Duration	Intervention	Comparator	Primary outcome(s)	Key results
Akhondzadeh et al. (2010) [14]	DB-RCT	46	16 weeks	Saffron 30 mg/day	Placebo	ADAS-Cog, CDR-SB	Significant improvement vs. placebo (ADAS-Cog Δ: 14.9 vs. 2.0; p<0.0001)
Akhondzadeh et al. (2010) [15]	DB-RCT	54	22 weeks	Saffron 30 mg/day	Donepezil 10 mg/day	ADAS-Cog, CDR-SB	Comparable efficacy; fewer adverse events with saffron
Farokhnia et al. (2014) [16]	DB-RCT	68	52 weeks	Saffron 30 mg/day	Memantine 20 mg/day	SCIRS, FAST	No significant difference (p=0.38); equal tolerability
Ayati et al. (2020) [17]	Meta-analysis	4 RCTs	Varied	Saffron 30 mg/day	Placebo/Medications	ADAS-Cog, CDR-SB, ADL	Saffron > placebo; non-inferior to donepezil/memantine; well tolerated

In a 16-week, double-blind, placebo-controlled RCT, Akhondzadeh et al. (2010) evaluated the efficacy of saffron in patients with mild-to-moderate AD (n=46). Participants were randomized to receive either 30 mg/day of saffron or placebo. The primary outcome was change in the Alzheimer’s Disease Assessment Scale-Cognitive Subscale (ADAS-Cog) score. The saffron group showed significantly greater improvement than placebo (mean ADAS-Cog reduction: 14.9 vs. 2.0; p<0.0001), along with improvements in Clinical Dementia Rating-Sum of Boxes (CDR-SB). Adverse events were mild and included dizziness and dry mouth, with no serious adverse events reported [14].

In a more direct comparison, Akhondzadeh et al. (2010) conducted a 22-week, double-blind, multicenter RCT (n=54) comparing saffron (30 mg/day) to donepezil (10 mg/day). Both groups exhibited significant improvements in cognitive function over time, with no statistically significant difference between them on ADAS-Cog or CDR-SB scores (p>0.05). Importantly, the donepezil group experienced more frequent adverse events, particularly nausea and vomiting, whereas the saffron group was better tolerated overall [15].

Farokhnia et al. (2014) extended the evidence base by examining saffron in the later stages of AD. In this 12-month, double-blind RCT, patients with moderate-to-severe AD (n=68) with Mini-Mental State Examination (MMSE) scores between 8 and 14 were randomized to receive either saffron extract (30 mg/day) or memantine (20 mg/day). The primary outcomes were changes in the Severe Cognitive Impairment Rating Scale (SCIRS) and the Functional Assessment Staging Tool (FAST). Both groups showed statistically similar improvements (p=0.38 for SCIRS and p=0.87 for FAST), with no significant treatment-by-time interaction (p=0.08). Adverse events occurred at similar frequencies in both groups, indicating comparable tolerability [16].

A systematic review and meta-analysis by Ayati et al. (2020) synthesized data from four RCTs involving saffron for cognitive impairment and dementia. The review concluded that saffron significantly improved cognitive function compared to placebo (p<0.01) and was statistically non-inferior to conventional medications such as donepezil and memantine. Improvements in activities of daily living were observed but did not reach statistical significance. No serious adverse events were reported across the included trials, reinforcing saffron’s safety profile [17].

Notably, all the cognitive trials used a consistent saffron dose of 30 mg/day, and none required dosage titration. The duration of treatment ranged from 16 to 52 weeks, making the dataset relatively unique among herbal interventions in dementia for its longitudinal coverage. While the sample sizes were modest (n=46 to 68 per trial), the reproducibility of the findings across multiple independent teams lends credibility to the observed effects.

In summary, saffron has shown efficacy comparable to donepezil and memantine in improving cognitive function in AD, with a favorable side effect profile. These findings position saffron as a potentially viable therapeutic option, particularly for patients who cannot tolerate conventional medications due to gastrointestinal or cholinergic side effects.

Mechanisms of action

The therapeutic efficacy of saffron in mood and cognitive disorders is driven by the complementary actions of its bioactive compounds - primarily crocin, crocetin, and safranal - across multiple neurobiological pathways. These include monoaminergic regulation, cholinesterase inhibition, antioxidant activity, anti-inflammatory signaling, and neurotrophic support.

Monoaminergic Modulation

Crocin and safranal influence mood by modulating central neurotransmitters such as serotonin, dopamine, and norepinephrine. Saffron has been shown to inhibit serotonin reuptake and increase levels of these neurotransmitters in animal models, producing antidepressant effects comparable to fluoxetine [8]. These findings are consistent with clinical trials demonstrating saffron’s non-inferiority to SSRIs in mild-to-moderate depression.

Cholinesterase Inhibition

The cognitive enhancement observed may be partly attributed to saffron’s ability to inhibit acetylcholinesterase (AChE), thereby increasing synaptic availability of acetylcholine. This is similar to the mechanism of donepezil and rivastigmine. In vitro studies confirm that crocin and safranal inhibit AChE in a concentration-dependent manner by binding at the active site of the enzyme [18]. This cholinergic activity is particularly relevant in AD, where ACh deficiency underlies cognitive symptoms.

Antioxidant Activity

Crocin and crocetin, the bioactive compounds in saffron, demonstrate potent antioxidant properties that help mitigate oxidative stress, a shared underlying mechanism in both mood disorders and neurodegenerative diseases. Their antioxidative activity operates through several key mechanisms. First, crocin and crocetin act as direct scavengers of reactive oxygen species (ROS), neutralizing free radicals and thereby reducing cellular oxidative burden [19-21]. Second, they significantly reduce lipid peroxidation by lowering the levels of malondialdehyde (MDA), a key biomarker of membrane lipid damage [22]. Third, saffron enhances the body’s endogenous antioxidant defenses by increasing the activity of superoxide dismutase (SOD) and glutathione (GSH), both of which are vital for maintaining intracellular redox balance [19,20]. This multifaceted antioxidant profile is especially important, as oxidative damage has been strongly implicated in neuronal apoptosis, cognitive decline, and the pathophysiology of depression.

Anti-Inflammatory Effects

Saffron also exhibits significant anti-inflammatory effects by modulating key innate immune pathways implicated in neuroinflammation and neurodegeneration. One of the primary mechanisms involves the inhibition of nuclear factor-kappa B (NF-κB) [23,24]. In addition, both saffron and its constituent safranal have been shown to reduce the production of key pro-inflammatory cytokines, including TNF-α, IL-6, and IL-1β in various in vitro and in vivo models [25]. Saffron also targets the nucleotide-binding oligomerization domain-like receptor protein 3 (NLRP3) inflammasome pathway, where safranal inhibits the oligomerization of apoptosis-associated speck-like protein containing a caspase recruitment domain (ASC) and suppresses caspase-1 activation, effectively decreasing IL-1β cleavage and overall inflammasome activity [25]. Furthermore, saffron activates nuclear factor erythroid 2-related factor 2 (Nrf2), which upregulates the expression of genes with antioxidant and anti-inflammatory properties, offering a dual protective mechanism [24]. These anti-inflammatory actions are particularly relevant in the context of neurodegenerative diseases, where persistent low-grade inflammation and microglial activation contribute to neuronal damage and accelerate disease progression.

Neuroprotection and Synaptic Plasticity

Crocin exerts notable neuroprotective effects through its modulation of neurotrophic signaling pathways. It plays a key role in mitigating glutamate-induced excitotoxicity by reducing ROS production and lipid peroxidation, thereby protecting neurons from oxidative stress-induced apoptosis [26]. In addition to its antioxidative role, crocin enhances the expression of brain-derived neurotrophic factor (BDNF), a vital protein involved in synaptic plasticity, learning, and memory [27,28]. This effect is further supported by its activation of the cAMP response element-binding protein (CREB) pathway, which leads to the phosphorylation of CREB and subsequent transcription of BDNF and other neurogenic genes [27]. Moreover, both crocin and crocetin promote neurogenesis by facilitating the differentiation of neural stem cells into mature neurons, contributing to post-injury neuronal recovery and the preservation of cognitive reserve [29]. These mechanistic insights are consistent with saffron’s demonstrated clinical efficacy in AD and MCI, where synaptic degeneration and diminished BDNF levels are key contributors to disease progression.

Challenges and limitations

Despite its promise, saffron faces several barriers to widespread clinical adoption. One of the most pressing concerns is extract standardization. The concentration of crocin and safranal can vary widely based on the geographic origin, harvest season, and processing methods, making it difficult to establish consistent dosing guidelines [6]. Additionally, most existing RCTs evaluating saffron have small sample sizes and relatively short durations (typically 6-22 weeks), which limits our understanding of its long-term efficacy and safety.

There is also a lack of research exploring pharmacogenomic or metabolic predictors of the response to saffron. Future trials incorporating biomarkers and stratified patient data will be essential for integrating saffron into precision medicine frameworks.

Accessibility and cost-effectiveness

From a pharmacoeconomic perspective, saffron’s low side-effect profile and growing evidence base make it an attractive alternative or adjunct to conventional therapies, particularly in populations with poor medication tolerance. However, high-grade saffron remains costly to cultivate and produce, and limited regulatory approval may restrict insurance reimbursement and clinical adoption. Nonetheless, its cultural familiarity and natural origin may appeal to patients seeking integrative approaches to mental and cognitive health [6].

## Conclusions

Growing clinical and preclinical evidence suggests that saffron is a safe and potentially effective treatment for depression, anxiety, and cognitive decline, including mild cognitive impairment and AD. RCTs have demonstrated that saffron is comparable in efficacy to first-line antidepressants such as fluoxetine in patients with mild-to-moderate depression. In the cognitive domain, head-to-head trials of saffron with donepezil and memantine have found that it is non-inferior in improving cognitive outcomes such as ADAS-Cog and CDR-SB scores, with fewer gastrointestinal side effects. A meta-analysis further supports saffron’s statistically significant superiority to placebo and equivalence to cholinesterase inhibitors and NMDA antagonists, offering a viable alternative for patients who cannot tolerate standard medications.

Nonetheless, key gaps remain. Most trials to date are limited by small sample sizes, short durations, geographic concentration (primarily in Iran), and a lack of biomarker validation. Standardization of the saffron extract, dose optimization, and long-term safety evaluation are critical steps toward regulatory approval and clinical integration. Additionally, pharmacokinetic studies and precision medicine approaches, such as identifying biomarkers of treatment response, are needed to individualize therapy. If these challenges are addressed through larger, multicenter trials and mechanistic studies, saffron may transition from a traditional herbal remedy to an evidence-based adjunct or alternative in the treatment of mood and cognitive disorders. While saffron shows clinical promise, it should not yet replace first-line treatments, given the limitations of existing evidence.
